# Pertussis immunization during pregnancy: results of a cross-sectional study among Italian healthcare workers

**DOI:** 10.3389/fpubh.2023.1214459

**Published:** 2023-07-06

**Authors:** Francesca Licata, Marika Romeo, Gianfranco Di Gennaro, Emma Antonia Citrino, Aida Bianco

**Affiliations:** Department of Health Sciences, School of Medicine, University of Catanzaro “Magna Græcia”, Catanzaro, Italy

**Keywords:** healthcare workers, immunization, pertussis, pregnancy, Tdap, vaccination

## Abstract

**Background:**

This study aimed to assess whether Italian healthcare workers (HCWs) recommend the reduced antigen content tetanus-diphtheria-acellular pertussis vaccination (Tdap) to pregnant people, as well as what variables could predict their decision to advise and recommend immunization to pregnant people.

**Methods:**

This cross-sectional study took place between August 2021 and June 2022 in a sample of obstetricians-gynecologists, midwives, and primary-care physicians in two regions of Southern Italy. A self-administered questionnaire was used to gather the data.

**Results:**

The results showed 91.3% (379) of participants knew that receiving the Tdap vaccine during pregnancy protects against pertussis in both the expectant person and the newborn before active immunization. Only 68.9% (286) knew that the Tdap vaccination has to be administered during the third trimester of gestation. A small but still significant proportion of participants (14.7%) (61) believed that the potential risks of vaccines administered during pregnancy outweighed the benefits. An improvable proportion of HCWs regularly provided information [71.8% (298)] and recommended [81% (336)] Tdap vaccination to pregnant people. The strongest factors that drove HCWs to inform pregnant people about the Tdap vaccination were to be aware that vaccinating those in close contact with newborns is an effective strategy to prevent pertussis (OR: 2.38; 95% CI: 1.11–5.13) and that the Tdap vaccine is provided only in the third trimester of pregnancy (OR: 1.74; 95% CI: 1.06–2.86). Informing pregnant people about the possibility of receiving the Tdap vaccine during pregnancy (OR: 60.13; 95% CI: 23.50–153.8) was the strongest predictor of having recommended the Tdap vaccination during pregnancy.

**Conclusion:**

Educational and informative interventions to improve HCWs’ knowledge about the importance of the Tdap vaccine and their communication skills to properly counsel pregnant people are needed. Beyond vaccine recommendations, how well immunization strategies are implemented in real-world situations impacts vaccination uptake. Therefore, during regular care visits, expecting people must have easy access to vaccines. Prenatal immunizations should become common practice, and there should be no conceptual doubt about vaccinations among HCWs to safeguard pregnant people and their unborn children from vaccine-preventable diseases.

## Introduction

Evidence exists to indicate that maternal pertussis vaccination can reduce the risk of pertussis, hospitalization, or death among infants by between 69 and 95% (1). Pertussis can be deadly, especially in babies below 3 months of age (2). Therefore, vaccination campaigns among pregnant people have been introduced in many countries, including Italy, to protect newborns through the natural transmission of passive immunity (3, 4). The Italian Ministry of Health enacted a National Immunization Plan in which it is stated that reduced antigen content tetanus-diphtheria-acellular pertussis vaccine (Tdap) is recommended to be administered from the 27th to the 36th week of pregnancy (ideally at the 28th week) and at each pregnancy (5), to provide adequate protection of newborns. Nevertheless, immunization coverage among pregnant people remains below the recommended threshold of 95% (2). One potential reason for this could be found in vaccine hesitancy, defined as a delay in acceptance or refusal of vaccines, despite the availability of vaccination services (6). Hesitancy is then considered one of the most important global health issues by the World Health Organization (WHO) (7). Acceptance or rejection of vaccines among pregnant people may depend on many variables. Women can show hesitancy toward vaccines during pregnancy as a consequence of their vaccination reluctance in general or because of a lack of information, as well as previous negative individual experiences with vaccines that can strongly contribute to this phenomenon (8). Looking specifically at the Italian population, low vaccine knowledge among those with a low level of education appeared to be the most common determinant of low levels of vaccination uptake during pregnancy (9). However, considering that vaccine hesitancy is context-dependent (10), lack of information may represent just one of the reasons underlying the phenomenon. Hence, the contact and conversation between healthcare workers (HCWs) and parents when discussing parental vaccination concerns is not only widely acknowledged as being crucial in informing parents about vaccines but also in easing parental anxieties (11). Even vaccine-hesitant parents, in fact, consider HCWs as a trusted channel to address common doubts about vaccines (12). HCWs then, i.e., primary-care physicians (PCPs), pediatricians, gynecologist-obstetricians (OB-GYNs), and nurse-midwives, play an important role in providing clear information about vaccines and in addressing parents’ concerns (13). Therefore, considering the aforementioned data, it appears interesting to assess whether Italian HCWs recommend the Tdap vaccination to pregnant people, how valuable they consider their contribution in implementing vaccination uptake during pregnancy, and which attitudes influence their practices. We also decided to evaluate their degree of knowledge on the topic, trying to frame the southern Italy reality when it comes to enhancing immunization plans among pregnant people. Furthermore, seeing as healthcare operators’ point of view can be a resource in understanding which strategies might implement recommended vaccine uptake during pregnancy, part of the assessment has been dedicated to it.

## Materials and methods

### Study design and setting

The present study followed the Strengthening the Reporting of Observational Studies in Epidemiology (STROBE) reporting guidelines for observational studies (14). This cross-sectional study was conducted between August 2021 and June 2022 in a sample of OB-GYNs, midwives, and PCPs in two regions of southern Italy: Calabria and Sicily. A multi-stage sampling design was used. First, we selected using simple random sampling at two teaching hospitals and two tertiary care public hospitals. In addition, PCPs practicing in those regions were randomly selected from a publicly available frame.

### Data collection and study sample

Data were collected using a self-administered paper questionnaire distributed by trained medical staff.

Before starting to collect questionnaires, a letter was sent to the management staff of the selected hospital to explain the purposes of the study and obtain their written consent to carry out the survey in their institution. All participants were informed of the background, objectives, and privacy rules related to the survey. A signed informed consent form was obtained from all participants who agreed to participate in the study clarifying that anonymity and confidentiality of collected data were guaranteed. HCWs who declined to sign the informed consent were excluded from the study. We purposively recruited participants who met the following eligibility criteria: OB-GYN, PCP, or registered midwife and having a good command of Italian. The participants did not receive any form of payment or incentives for taking part in this study.

### Sample size

A minimum sample size of 368 was calculated using the Raosoft sample size calculator (15) providing a confidence level of 95% with a margin of error of 5%. The article by Kissin et al. (16) reported that mean response rates for similar surveys were 42.3%; therefore, to maximize the number of responses, 696 surveys were distributed.

### Questionnaire design

The questionnaire was developed after an extensive literature review (16–20). The questionnaire’s comprehensibility, clarity, and ease of administration use were evaluated using a pilot test (10 HCWs not included in the final sample). Minor refinements were made based on the feedback received from this phase. The final questionnaire used a combination of checkboxes and free text answers, which consisted of 17 items divided into five sections. It took approximately 10 min to complete all items. The first section of the questionnaire collected information about the sociodemographic and professional characteristics of the participants (four items, closed-ended with multiple answers and open-ended) including age, gender, professional specialty, and years in practice. The second section (three items with multiple answers “true, false, do not know”) investigated general knowledge about vaccinations during pregnancy. The third section (four items on a 5-point Likert scale, ranging from “strongly disagree” to “strongly agree”) tested attitudes and beliefs regarding the benefits and risks of vaccinating pregnant people. The fourth section (four items with multiple answers and open options) explored providers’ vaccination behaviors, whether they informed and advised pregnant people on Tdap vaccination, and also assessed strategies and interventions to increase uptake of vaccination during pregnancy. The last section (two items, closed-ended with multiple answers and open options) analyzed the sources of information on vaccination, the level of satisfaction with these different sources, and the need to receive additional information about recommended vaccination during pregnancy. This study received approval from the Calabria Center Local Human Research Ethics Committee (ID No. 275/2021/07/15).

### Statistical analysis

All collected variables were obtained by means and standard deviations when normally distributed. In cases of deviations from normality, medians and interquartile ranges were utilized. Categorical variables were expressed in percentages. Logistic regression models were developed to explore the role of potential predictors of the following outcomes of interest: having informed about the Tdap vaccination during pregnancy (no = 0; yes = 1) (Model 1) and having recommended the Tdap vaccine during pregnancy (no = 0; yes = 1) (Model 2). The following selected independent variables were included in both models: age in years (continuous), sex (male = 0; female = 1), profession (OB-GYNs = 0; PCPs = 1; 2 = midwife), number of years of practice (continuous), knowledge that Tdap vaccine administered during pregnancy protects only the expectant person (I do not know/true = 0; false = 1), in addition to knowledge that vaccinating pregnant people and those in close contact with newborns is an effective strategy to prevent pertussis (I do not know/false = 0; true = 1), and knowledge that Tdap vaccine is provided only in the third trimester of pregnancy (I do not know/false = 0; true = 1), belief that improving adherence to vaccinations in pregnancy is an efficient prevention strategy (Uncertain/strongly disagree/disagree = 0; Strongly agree/agree = 1), belief that the potential risks of vaccinations administered during pregnancy are greater than the benefits (Uncertain/agree/strongly agree = 0; strongly disagree/disagree = 1), belief that vaccinating pregnant people against pertussis is an effective way to reduce the risk of pertussis in the unborn child (Uncertain/strongly disagree/disagree = 0; Strongly agree/agree = 1), and belief that providing detailed information about the effectiveness and safety of vaccinations is a useful strategy to improve vaccine uptake in pregnancy (Uncertain/strongly disagree/disagree = 0; Strongly agree/agree = 1). In Model 2, the variable informing pregnant people about the possibility of receiving the Tdap vaccine during pregnancy (never/rarely/sometimes = 0; often/always = 1) was also included. The Hosmer and Lemeshow test assessed the goodness of fit of the logistic model and visual investigation of the lowess curve fitting liner predictor (log-odds) values by Pearson’s standardized residuals. The statistical significance level was fixed at a value of p of <0.05. Adjusted odds ratio (OR) and 95% confidence interval (CI) were calculated. Statistical analysis was developed using the STATA software program, version 16.1 (21).

The dataset was deposited in the Mendeley Data repository (doi: 10.17632/7x785tzhyh.2).

## Results

### Participants’ demographics

Of the eligible 696 HCWs approached, 415 agreed to participate for a response rate of 59.6%. The study sample consisted of 415 HCWs, including OB-GYNs (64.8%), midwives (21.7%), and PCPs (13.5%) with an average age of 42.8 years (±11.3). Of the participants, 60.5% were female and 39.5% were male. The mean number of years in practice was 14 (± 11.5). Table 1 shows participant characteristics.

**Table 1:** Characteristics of the study population (415 respondents).

	*N*	%	Mean ± SD
Age, in years			42.8 ± 11.3
Sex			
Male	164	39.5	
Female	251	60.5	
Professionals			
OB-GYN	269	64.8	
Midwife	90	21.7	
PCP	56	13.5	
Number of years of practice			13.4 ± 11.6

OB-GYN, obstetrician-gynecologist; PCP, primary care physician.

### Healthcare workers knowledge of vaccinations and attitudes toward vaccines during pregnancy

HCWs’ knowledge and attitudes toward recommended vaccinations during pregnancy and vaccine-preventable diseases (VPDs) were investigated. The results are shown in Table 2. Almost all of the participants (91.3%) knew that the Tdap vaccine administered during pregnancy protects the expectant person and the newborn; 87.5% of the sample was aware that vaccinating those in close contact with newborns (i.e., cocoon strategy) is an effective way of preventing pertussis in children during their first months of life. Lastly, even though more than half (68.9%) of the respondents correctly affirmed that the Tdap vaccine is provided only in the third trimester of pregnancy, a good percentage (31.1%) answered incorrectly.

**Table 2:** HCWs’ level of knowledge and attitudes toward recommended vaccinations during pregnancy.

Knowledge statements (415 respondents)	Correct	
	*N*	%
Tdap vaccine administered during pregnancy protects only expectant people (false)	379	91.3
In addition to vaccination during pregnancy, vaccinating those in close contact with newborns is an effective strategy to prevent pertussis (true)	363	87.5
Tdap vaccine is provided only in the 3rd trimester of pregnancy (true)	286	68.9

Attitudes statements (415 respondents)	Strongly disagree/disagree		Uncertain		Strongly agree/agree	
	*N*	%	*N*	%	*N*	%
Improving adherence to vaccinations in pregnancy is an efficient prevention strategy	8	1.9	8	1.9	**399**	**96.1**
The potential risks of vaccinations administered during pregnancy are greater than the benefits	**354**	**85.3**	16	3.9	45	10.8
Vaccinating pregnant people against pertussis is an effective way to reduce the risk of pertussis in the unborn child	15	3.6	30	7.2	**370**	**89.2**
Providing detailed information about the effectiveness and safety of vaccinations is a useful strategy to improve vaccine uptake in pregnancy	2	0.5	13	3.1	**400**	**96.4**

HCWs, healthcare workers; Tdap, Tetanus toxoid, reduced diphtheria toxoid, and acellular pertussis.

In bold are number and percentages referring to positive attitudes.

Almost the entire sample (96.1 and 96.4%, respectively) believed that improving adherence to vaccinations in pregnancy is an efficient prevention strategy, and providing detailed information about the effectiveness and safety of vaccinations is a useful strategy to improve vaccine uptake in pregnancy. In total, 85.3% of the interviewed considered that the potential risks of vaccines administered during pregnancy are lesser than the benefits. Furthermore, 89.2% supposed that vaccinating pregnant people against pertussis is an effective way to reduce the risk of infection in the unborn child.

### Healthcare workers behaviors about vaccinations recommended during pregnancy

Almost three quarters (71.8%) of the interviewed HCWs often/always provided information about Tdap vaccination to pregnant people, but, on the other hand, 20.8% of OB-GYNs, 32.1% of PCPs, and 47.8% of midwives affirmed they never or rarely or sometimes do it; moreover, 81% of the sample often/always recommended pregnant people to get vaccinated for Tdap during pregnancy. Among those who recommended vaccination never or rarely or sometimes, 9.7% were OB-GYNs, 30.4% were PCPs, and 40% were midwives (Table 3). The results of the multiple logistic regression analysis (Model 1 in Table 4) indicated that the strongest factor that had driven HCWs to inform pregnant people about the Tdap vaccination was having good knowledge about it, in particular, knowing that vaccinating pregnant people and those in close contact with newborns is an effective strategy to prevent pertussis (OR: 2.38; 95% CI: 1.11–5.13) and that the Tdap vaccine is provided only in the third trimester of pregnancy (OR: 1.74; 95% CI: 1.06–2.86). Among the subjects who often or always recommended vaccination, 93.7% stated that the Tdap vaccine must be recommended to all pregnant people, 2.9% to women with high-risk pregnancies, 2.3% to women with chronic diseases, and 1.1% to HIV+ women. Informing pregnant people about the possibility of receiving the Tdap vaccine during pregnancy (OR: 60.13; 95% CI: 23.50–153.8) increased almost 60-fold the odds of having recommended Tdap vaccination during pregnancy. Similarly, believing that improving adherence to vaccinations during pregnancy is an efficient prevention strategy (OR: 5.38; 95% CI: 1.06–27.35) is indipendently associated with having recommended Tdap vaccination. Otherwise, a negative association was shown for participants who were PCPs (OR: 0.32; 95% CI: 0.11–0.98) or midwives (OR: 0.20; 95% CI: 0.08–0.55) (Model 2 in Table 4). The most common reasons cited for not recommending vaccination against pertussis included the belief that it was outside the scope of their practice (51.3%) and, among those, 90% were midwives and 10% were PCPs; vaccine hesitancy among pregnant people (35.9%) and, among those, 57.2% were midwives, 35.7% were Obs, and 7.1% were PCPs; and lack of knowledge (31.3%) and, among those, 84.6% were midwives and 15.4% were PCPs (Table 3). On the other hand, HCWs indicated the following as possible strategies to improve vaccine uptake in pregnancy: offering vaccinations during regular care visits in pregnancy (58.8%), informing and educating expectant people about the availability, effectiveness, and safety of vaccinations during pregnancy (55.9%), improving accessibility to vaccination services (e.g., flexible schedules and weekend vaccination sessions) (44.3%), making a vaccine clinic available at the hospital (38.7%), allowing midwives to vaccinate pregnant people (21.1%), and reminding/offering vaccination through text messages or emails (16.5%) (Table 5).

**Table 3:** HCWs’ behaviors about Tdap vaccination during pregnancy.

Statements	Never/rarely/sometimes		Often/always	
	*N*	%	*N*	%
Informing pregnant people about the possibility of receiving Tdap vaccine during pregnancy (415)	117	28.2	298	71.8
OB-GYNs (269)	56	20.8	213	79.2
PCPs (56)	18	32.1	38	67.9
Midwives (90)	43	47.8	47	52.2
Recommending pregnant people to get vaccinated for Tdap during pregnancy (415)	79	19	336	81
OB-GYNs (269)	26	9.7	243	90.3
PCPs (56)	17	30.4	39	69.6
Midwives (90)	36	40	54	60

Reasons for the non-recommendation of Tdap vaccination during pregnancy								
Statements*	Total sample (39)		OB-GYN (269)		PCPs (56)		Midwives (90)	
	*N*	%	*N*	%	*N*	%	*N*	%
Outside the scope of practice	20	51.3	-		2	10	18	90
Vaccine hesitancy among pregnant people	14	35.9	5	35.7	1	7.1	8	57.2
Lack of knowledge	13	33.3	-		2	15.4	11	84.6
Lack of time	7	18	-		3	42.9	4	57.1
Fear of adverse events	3	7.7	1	33.3	1	33.3	1	33.3
Skepticism about the effectiveness of vaccines	1	2.6	-		1	100	-	

HCWs, Healthcare workers; Tdap, reduced antigen content tetanus-diphtheria-acellular pertussis vaccine; OB-GYNs, obstetricians-gynecologists; PCPs, primary-care physicians.

* Multiple responses allowed.

**Table 4:** Results of the regression model for potential determinants of the outcomes of interest.

Model 1: Outcome: having informed about Tdap vaccination during pregnancy. Log-likelihood = −218.10082; Prob > chi2 < 0.001; Obs = 415		
Variables	OR	95% CI
Knowledge that vaccinating pregnant people and those in close contact with newborns is an effective strategy to prevent pertussi		
I do not know/false*	1.00	
True	2.38	1.11–5.13
Knowledge that Tdap vaccine is provided only in the 3^rd^ trimester of pregnancy		
I do not know/false*	1.00	
True	1.74	1.06–2.86
Professionals		
OB-GYN*	1.00	
Midwife	0.54	0.28–1.03
PCP	0.66	0.32–1.37
Knowledge that Tdap vaccine administered during pregnancy protects only the expectant person		
I do not know/true*	1.00	
False	2.10	0.91–4.86
Belief that improving adherence to vaccinations in pregnancy is an efficient prevention strategy		
Uncertain/strongly disagree/disagree*	1.00	
Strongly agree/agree	2.76	0.79–9.58
Age in years, continuous	1.06	0.98–1.15
Number of years of practice, continuous	0.95	0.88–1.03
Belief that vaccinating pregnant people against pertussis is an effective way to reduce the risk of pertussis in the unborn child		
Uncertain/strongly disagree/disagree*	1.00	
Strongly agree/agree	1.39	0.66–2.93
Belief that the potential risks of vaccinations administered during pregnancy are greater than the benefits		
Uncertain/agree/strongly agree*	1.00	
Disagree/strongly disagree	1.34	0.69–2.59
Belief that providing detailed information about the effectiveness and safety of vaccinations is a useful strategy to improve vaccine uptake in pregnancy		
Uncertain/strongly disagree/disagree*	1.00	
Strongly agree/agree	0.75	0.20–2.85
Sex		
Male*	1.00	
Female	0.91	0.54–1.52

Model 2: Outcome: having recommended Tdap vaccination during pregnancy. Log-likelihood = −96.788919; Prob > chi2 < 0.001; Obs = 415		
Variables	OR	95% CI
Informing pregnant people about the possibility of receiving Tdap vaccine during pregnancy		
No*	1.00	
Yes	60.13	23.50–153.87
Professionals		
OB-GYN*	1.00	
Midwife	0.20	0.08–0.55
PCP	0.32	0.11–0.98
Belief that improving adherence to vaccinations in pregnancy is an efficient prevention strategy		
Uncertain/strongly disagree/disagree*	1.00	
Strongly agree/agree	5.38	1.06–27.35
Belief that providing detailed information about the effectiveness and safety of vaccinations is a useful strategy to improve vaccine uptake in pregnancy		
Uncertain/strongly disagree/disagree*	1.00	
Strongly agree/agree	6.15	0.90–41.90
Sex		
Male*	1.00	
Female	1.60	0.69–3.70
Belief that the potential risks of vaccinations administered during pregnancy are greater than the benefits		
Uncertain/agree/strongly agree*	1.00	
Disagree/strongly disagree	1.67	0.64–4.35
Knowledge that vaccinating pregnant people and those in close contact with newborns is an effective strategy to prevent pertussis		
I do not know/false*	1.00	
True	1.73	0.55–5.45
Knowledge that Tdap vaccine administered during pregnancy protects only the expectant person		
I do not know/true*	1.00	
False	0.77	0.21–2.76
Belief that vaccinating pregnant people against pertussis is an effective way to reduce the risk of pertussis in the unborn child		
Uncertain/strongly disagree/disagree*	1.00	
Strongly agree/agree	0.85	0.25–2.90
Age in years, continuous	0.98	0.88–1.09
Knowledge that Tdap vaccine is provided only in the 3^rd^ trimester of pregnancy		
I do not know/false*	1.00	
True	0.97	0.44–2.17
Number of years of practice, continuous	1.01	0.90–1.11

Tdap, reduced antigen content tetanus-diphtheria-acellular pertussis vaccine; OB-GYNs, obstetricians-gynecologists; PCPs, primary-care physicians.

* Reference category.

**Table 5:** Possible strategies to improve vaccine uptake in pregnancy reported by HCWs.

Statements*	Total sample (413)		Informing about Tdap vaccination (297)^#^		Recommending about Tdap vaccination (335)^$^	
	*N*	%	*N*	%	*N*	%
Offering vaccinations during regular care visits in pregnancy	243	58.8	185	62.3	215	64.2
Informing and educating expectant people about the availability, effectiveness, and safety of vaccinations during pregnancy	231	55.9	162	54.5	186	55.5
Improving accessibility to vaccination services (e.g., flexible schedules, weekend vaccination sessions)	183	44.3	137	46.1	156	46.6
Making a vaccine clinic available at the hospital	160	38.7	128	43.1	146	43.6
Midwives vaccinating pregnant people	87	21.1	64	21.5	68	20.3
Reminding/offering vaccination through text messages or email	68	16.5	54	18.2	61	18.2

* Multiple responses allowed.

# Eligible HCWs were those who reported having often/always informed pregnant people about Tdap vaccinations.

$ Eligible HCWs were those who reported having often/always recommended pregnant people about Tdap vaccinations.

### Sources of information

Regarding the preferred sources of information used by HCWs, the highest percentage (85.4%) was represented by conferences with a degree of satisfaction equal to 74.6%, while the lowest one (0.5%) was the Internet. In addition to the aforementioned results, it was found that almost two-thirds (60.2%) of the sample declared the need to have more information about recommended vaccinations during pregnancy.

## Discussion

Despite a monitoring system is not yet in place at the national level, Tdap coverage during pregnancy seems to be suboptimal in Italy against recommendations (22). Since the single best predictor of vaccination among pregnant people is a strong HCWs’ recommendation coupled with an offer of vaccination (23–26), we hypothesized that HCWs who are knowledgeable about the importance of vaccination in pregnancy and have positive attitudes toward it are more likely to persuade pregnant people to accept the vaccine, as previously demonstrated in other contexts (27, 28). With this in mind, the findings of the present study provide up-to-date insight into immunization needed during pregnancy that will aid in improving HCWs’ counseling techniques and assist them in their crucial role of guiding and supporting the decisions of pregnant people regarding the Tdap vaccine.

Four important points emerged from the study. In the first place, the results showed HCWs’ lack of confidence and understanding about the proper time frame during which to administer the Tdap vaccine during pregnancy. The study’s findings revealed that more than two-thirds of the sample did not know that pregnant people can only receive the Tdap vaccine during the third trimester of pregnancy. This is of concern, considering that to enhance maternal antibody response and passive antibody transfer to the fetus, the administration should take place between 27 and 36 weeks of gestation, ideally around the 28th week. To ensure that every infant obtains the best possible protection against pertussis at birth and until the third dose is administered, pregnant people should be advised to get vaccinated during the specific abovementioned time frame.

The second important and alarming result was that HCWs’ perceptions of the benefits and risks of immunizations for unborn children and their mothers did not seem to be consistent with the desired outcome among this population. In the survey, a small but still significant proportion of HCWs claimed that the possible risks of immunizations given during pregnancy outweigh the benefits. In addition, some of the responders did not consider the Tdap vaccine as an effective strategy to reduce the risk of pertussis in the unborn child. On the contrary, the WHO SAGE Committee, the Centers for Disease Control (CDC), the American College of Obstetricians and Gynecologists (ACOG), and the British Joint Committee on Vaccination (JCVI) all contributed to state that maternal Tdap vaccination gives babies passive protection while also helps expectant people avoid contracting and spreading pertussis to their children. Given serious and sometimes life-threatening complications among babies younger than 1 year of the infection (29), pregnancy is the best time to immunize and to achieve protection for both the expectant person and the fetus from VPDs.

In Italy, a strong inverse link between hospitalization rates and vaccination rates, especially for infants under 1 year old, was shown. Moreover, most side effects from Tdap vaccination during pregnancy are mild or moderate and self-resolving, and no safety signals among pregnant people or their babies after Tdap vaccination were found. On the other hand, nearly one-third of babies younger than 1 year who get pertussis needing care in the hospital, and 1 out of 100 babies who get treated in the hospital die (29, 30). Therefore, according to research (31), increasing HCWs’ awareness of pertussis infection and the effectiveness and safety of vaccination may boost their likelihood of recommending the Tdap vaccine.

Third, almost one-third of the respondents reported they did not counsel or notify expectant people about the potential of obtaining the Tdap vaccine during pregnancy, missing an opportunity for immunization. In this situation, immunizations are not seen as a top priority, especially if the HCW staff has not made a clear recommendation for them, in both the pre-service and in-service phases. The fact that pregnant people cannot rely on HCWs to inform them about immunizations during pregnancy raises concerns since they must be aware of the possibility of receiving the Tdap vaccine to choose whether to get vaccinated or not. Poor knowledge and concern about vaccine safety are displayed as the main reasons for vaccine hesitancy among pregnant people (32). The tendency to associate serious side effects with vaccines and the underestimation of risks of severe illness during pregnancy are important drivers of the phenomenon of vaccine hesitancy among pregnant people (32). The finding that informing pregnant people about the possibility of receiving the Tdap vaccine is the strongest predictor of having recommended Tdap vaccination during pregnancy underlines the crucial role of accurate information about maternal immunization. Hence, lack of advise or the fact that the OB-GYN discourages them from getting the Tdap vaccine might lead pregnant people not to get vaccinated (33). Each and every part of the healthcare system needs to be comfortable with and in charge of informing and counseling individuals about the vaccines that are recommended during pregnancy. As evidenced by the fourth significant finding, the most often cited justifications for not advising Tdap immunization during pregnancy were that HCWs considered it outside the scope of their practice, or they accepted vaccine hesitancy during pregnancy as a non-modifiable factor. Therefore, responsibility for individual education should fall especially on the HCWs’ staff, and if HCWs do not stock or administer vaccines in the office, it is important to provide a referral to another immunization provider, making sure that everyone who needs immunization receives it. In the context of the study findings, midwives and PCPs seem to be the HCWs who deserve greater attention since they believed that recommending vaccination against pertussis was outside the scope of their practice. Among the HCWs, midwives and PCPs represent the first-line healthcare providers who have several interactions with pregnant people (34). However, in Italy, the role of those HCWs as reliable resources for expectant people counseling is largely neglected. As such, the need for adequate training to ensure proper management of vaccination during pregnancy is essential. Brief vaccine communication skills training for PCPs and midwives that include helpful advice on how to effectively communicate information in a health context could improve the uptake of maternal immunization (35). However, despite the benefits of maternal pertussis vaccination, implementation has not yet become standard practice, and it is frequently severely constrained because of structural and socio-cognitive barriers (36). Pregnant people expect HCWs who routinely follow them during pregnancy to provide information on the effectiveness and safety of Tdap vaccination and to act as trustworthy interlocutors for doubts, questions, and explanations. Therefore, a start in the right direction would be more HCW involvement in decision-making processes relating to vaccination recommendations and policies that they are actively implementing, with HCWs getting vaccination training to be knowledgeable about and confident in their ability to conduct the maternal immunization program, which will increase the uptake of vaccines during pregnancy and after birth. It is reasonable then to consider the latter as a contributing factor to the perceived lack of responsibility.

### Limitations

The interpretation of the study findings should consider some limitations. The first limitation attains the possibility of desirability bias as the data were self-reported, but the direct observation was not feasible due to the expense involved and the risk of producing observation bias. Nevertheless, assurance of anonymity and confidentiality of the data in the survey minimizes the probability of this bias. Second, the response rate is lower than the desired, but it could be considered satisfactory for surveys conducted on HCWs (37–39), suggesting that non-response bias had no substantial effect on the results. Furthermore, the data were collected in two Italian regions, which might not represent Italian HCWs but may represent the southern part of Italy.

## Conclusion

The advice given by HCWs about immunization during pregnancy must be backed up by recent, reliable scientific evidence. Beyond vaccine recommendations, how well immunization strategies are implemented in real-world situations impacts vaccination uptake. Therefore, during regular care visits, expecting people must have easy access to vaccines. Prenatal immunizations should become common practice, and there should be no conceptual doubt about vaccinations among HCWs to safeguard pregnant people and their unborn children from VPDs.

## Author note

The preliminary results (on 94 out of 415 HCWs) of this study were presented as an E-Poster at the 15th European Public Health Conference held in Berlin, Germany in November 2022 and published in the European Journal of Public Health, Volume 32, Supplement 3, 2022.

## Data availability statement

The dataset presented in this study can be found in online repository. The names of the repository and accession number can be found at: Mendeley Data repository (doi: 10.17632/7x785tzhyh.2).

## Ethics statement

The studies involving human participants were reviewed and approved by the Calabria Centre Local Human Research Ethics Committee (ID No. 275/2021/07/15). The participants provided their written informed consent to participate in this study.

## Author contributions

AB, FL, and MR participated in the conceptualization and design of the study. MR contributed to the data collection. MR, FL, GDG, and EAC contributed to the data analysis and interpretation. MR, FL, and EAC contributed to the preparation of the first draft of the manuscript. AB was responsible for funding acquisition and resource provision, the principal investigator, designed the study, coordinated and supervised data collection, was responsible for the statistical analysis and interpretation, and wrote the final article. All the authors have given final approval of the version to be published and agreed to be accountable for all aspects of the study.

## Funding

This study was supported by a grant from Sanofi.

## Conflict of interest

The authors declare that the research was conducted in the absence of any commercial or financial relationships that could be construed as a potential conflict of interest.

## Publisher’s note

All claims expressed in this article are solely those of the authors and do not necessarily represent those of their affiliated organizations, or those of the publisher, the editors and the reviewers. Any product that may be evaluated in this article, or claim that may be made by its manufacturer, is not guaranteed or endorsed by the publisher.
